# Disparities in colorectal cancer screening in New York City: An analysis of the 2014 NYC Community Health Survey

**DOI:** 10.1002/cam4.2084

**Published:** 2019-03-07

**Authors:** Neelesh Rastogi, Yuhe Xia, John M. Inadomi, Simona C. Kwon, Chau Trinh‐Shevrin, Peter S. Liang

**Affiliations:** ^1^ NYU Langone Health New York New York; ^2^ University of Washington School of Medicine Seattle Washington; ^3^ VA New York Harbor Health Care System New York New York; ^4^ Present address: Icahn School of Medicine at Mount Sinai New York New York

**Keywords:** colorectal cancer, disparities, New York City, race/ethnicity, screening

## Abstract

**Background & Aims:**

Disparities in colorectal cancer (CRC) screening uptake by race/ethnicity, socioeconomic status, and geography are well documented. We sought to further characterize the relationship between sociodemographic factors and up‐to‐date colonoscopy use in a diverse urban center using the 2014 New York City Community Health Survey (NYCCHS).

**Methods:**

We examined overall colonoscopy uptake by race/ethnicity—with a particular interest in Asian and Hispanic subgroups—and used weighting to represent the entire 2014 NYC adult population. We also evaluated the association between 10 sociodemographic variables (age, sex, race/ethnicity, birthplace, home language, time living in the US, education, employment, income, and borough of residence) and colonoscopy use using univariable and multivariable logistic regression models.

**Results:**

Up‐to‐date colonoscopy uptake was 69% overall with reported differences by racial/ethnic group, ranging from 44%‐45% for Mexicans and Asian Indians to 75% for Dominicans. In the multivariable regression model, colonoscopy use was associated with age greater than 65 years, Chinese language spoken at home, and not being in the labor force. Lower colonoscopy use was associated with living in the US for less than 5 years, Asian Indian language spoken at home, lower income, and residing outside of Manhattan.

**Conclusions:**

Among New Yorkers older than age 50, up‐to‐date colonoscopy use varied significantly by race/ethnicity, especially in Asian and Hispanic subgroups. Recent immigrants, low‐income groups, and those living outside of Manhattan were significantly less likely to receive CRC screening. Targeted interventions to promote CRC screening in these underserved groups may improve overall screening uptake.

## 1 INTRODUCTION

Screening has been definitively shown to reduce colorectal cancer (CRC) incidence and mortality.^1^, ^2^, ^3^, ^4^ CRC screening uptake in the United States (US) has steadily increased over time, from 34% nationally in 2000 to 62% in 2015.^5^ In New York City (NYC), the most populous city in the US with a population of 8.6 million, 57% of residents are racial/ethnic minorities, 49% speak a language other than English at home, and 20% live in poverty.^6^ The Citywide Colon Cancer Control Coalition (C5) was formed in 2003 with the purpose of increasing screening as a top public health issue. C5 primarily focused on colonoscopy, which is the predominant screening modality used in the US.^7^ Special initiatives were undertaken to increase screening in geographically and linguistically undeserved populations. These included a public health detailing program in Manhattan (Harlem), the Bronx, and Brooklyn in 2004 and 2008, as well as more recent tailored multimedia and provider outreach in neighborhoods with high concentrations of Russian and Chinese speakers. Under the leadership of the NYC Department of Health and Mental Hygiene (DOHMH), C5 initiatives saw screening colonoscopy uptake increase from 42% in 2003 to nearly 70% in 2013.^7^ In addition, previously observed racial/ethnic disparities in screening among non‐Hispanic whites (NHW), non‐Hispanic blacks (NHB), Asians, and Hispanics had disappeared by 2013.^8^ These findings stood in contrast to national data, where disparities in CRC screening by race/ethnicity, socioeconomic status, and geography have been well‐documented.^9^, ^10^, ^11^, ^12^, ^13^ We sought to conduct an in‐depth analysis of the relationship between sociodemographic factors and CRC screening using the 2014 New York City Community Health Survey (NYCCHS).^14^ We hypothesized that disaggregating Asian and Hispanic participants into ethnically distinct subgroups may reveal important differences in screening. Furthermore, despite closing the screening gap by race/ethnicity, disparities may still persist in domains such as socioeconomic status, acculturation, and health beliefs.

## 2 METHODS

### 2.1 Study population and design

We performed a cross‐sectional analysis using the 2014 NYCCHS, a telephone survey that has been administered annually by the New York City DOHMH since 2002. The survey samples approximately 10 000 randomly selected adults aged 18 years and older each year who reside in the five New York City boroughs (Manhattan, Brooklyn, Queens, Bronx, Staten Island). The participant cooperation rate in 2014 was 88.9%. Interviews were conducted in English, Spanish, Russian, Mandarin, and Cantonese.^14^ We focused on the subset of participants aged 50 years and older, for whom routine CRC screening is recommended. The outcome of interest was up‐to‐date CRC screening. Consistent with previous research on CRC screening using this dataset,^15^, ^16^ participants who indicated that they received a colonoscopy within the previous 10 years were considered to be up‐to‐date with CRC screening. We extracted individual‐level data on 10 sociodemographic variables of interest: age, sex, race/ethnicity, birthplace, primary language spoken at home, time living in the US, highest level of education attained, income relative to the federal poverty line, employment status, and borough of residence. Asian and Hispanic race/ethnicity were of particular interest and we collected data on disaggregated subgroup status (e.g. Chinese, Asian Indian, Puerto Rican, etc.).

### 2.2 Study outcomes

We calculated up‐to‐date CRC screening uptake by colonoscopy by race/ethnicity in 2014, including among disaggregated Asian and Hispanic subgroups. We also determined sociodemographic predictors of up‐to‐date screening. The study (18‐00012) was approved by the NYU School of Medicine Institutional Review Board.

### 2.3 Statistical analysis

Survey results were weighted using iterative proportional fitting to be representative of the entire New York City adult population.^14^ Weighting adjusts for the respondent's age, sex, race/ethnicity, neighborhood, and probability of selection. Risk estimates and confidence intervals were calculated using logistic regression models. For predictors of screening, we did not restrict entry into the multivariable logistic regression model based on any significance criteria from the univariable analysis. We used the aggregate Asian and Hispanic racial/ethnic subgroups in the multivariable regression model due to small sample sizes in some subgroups. Only birthplace was excluded from the final model because of multicollinearity with time living in the US. Results were considered statistically significant if the two‐sided *P* value was less than 0.05. All statistical analysis was performed using SAS software (SAS Institute Inc., Cary, NC).

## 3 RESULTS

### 3.1 Study population

Table 1 presents the sociodemographic features of the study population and weighted percentages. A total of 4190 individuals were included in the analysis. Of these, 1729 (42.7%) were men and 2461 (57.3%) were women. With respect to race/ethnicity, 1845 (43.9%) self‐identified as NHW, 836 (21.5%) as NHB, 1065 (23.4%) as Hispanic, 373 (10.0%) as Asian/Pacific Islander, and 71 (1.3%) as Other. Of the study population, 1966 individuals (52.0%) were foreign born.

**Table 1:** Demographic characteristics of the 2014 NYCCHS

Variable	n	% (weighted)^a^
Age		
50‐64	2277	60.6
65 and older	1913	39.4
Sex		
Male	1729	42.7
Female	2461	57.3
Race/ethnicity		
White, non‐Hispanic	1845	43.9
Asian/Pacific‐Islander	373	10.0
Black	836	21.5
Hispanic	1065	23.4
Other, non‐Hispanic	71	1.3
Asian/Hispanic subgroups		
Puerto Rican	373	9.2
Cuban	27	0.7
Dominican	350	8.6
Mexican	34	0.8
Central/South American	226	5.5
Other Hispanic	27	0.7
Chinese	271	6.7
Asian Indian	50	1.2
Filipino	22	0.5
Other Asian^b^	23	0.4
Birthplace		
United States	2206	48.0
Europe	314	9.0
Asia	379	10.0
Africa	53	1.4
Caribbean	875	22.2
Central/South America	305	8.1
Other	40	1.5
Home language		
English	2849	65.9
Spanish	769	17.9
Russian	161	4.9
Chinese	258	6.4
Indian	12	0.7
Other	133	4.1
Time living in US		
Born in US	2207	48.0
Lived in US less than 5 years	67	1.7
Lived in US 5‐9 years	82	2.2
Lived in US 10+ years	1818	48.1
Education		
College Graduate	1663	32.9
Some College	753	19.8
High school Graduate or less	1754	47.3
Employment status		
Unemployed	247	6.2
Employed	1767	43.1
Not in labor force	2155	50.7
Income relative to poverty line		
>200% of poverty line	2278	51.9
<200% of poverty line	1912	48.2
Borough of residence		
Manhattan	1136	19.9
Borough other than Manhattan	2054	80.1

a For each variable, except for Asian/Hispanic subgroups, weighted percentages total 100%.

b Korean, Japanese, Vietnamese, and Other.

### 3.2 CRC screening by race/ethnicity

Table 2 presents the weighted up‐to‐date CRC screening uptake in the overall population and in various disaggregated racial/ethnic groups. Screening uptake was 69.3% overall, 70.4% for NHW, and 68.2% for NHB. Among Hispanic subgroups, uptake ranged from 44.4% for Mexicans, to 75.3% for Dominicans, to 82.5% for Other Hispanics. Among Asian subgroups, uptake ranged from 29.0% for Other Asians (including Korean, Japanese, and Vietnamese), to 45.1% for Asian Indians, to 70.4% for Chinese. Compared with NHW, Other Asians (OR 0.17, 95% CI 0.05‐0.55), Asian Indians (OR 0.34, 95% CI 0.16‐0.76), and Mexicans (OR 0.34, 95% CI 0.15‐0.77) had significantly lower CRC screening uptake.

**Table 2:** Colonoscopy uptake by race and ethnicity

Race/ethnicity	% with Up‐to‐date Colonoscopy	OR (95% CI)
White, non‐Hispanic	70.4	Ref
Black, non‐Hispanic	68.2	0.90 (0.69‐1.17)
Puerto Rican	72.3	1.09 (0.77‐1.56)
Cuban	63.9	0.74 (0.26‐2.14)
Dominican	75.3	1.28 (0.87‐1.88)
Mexican	44.4	**0.34 (0.15‐0.77)**
Central or South American	70.5	1.00 (0.67‐1.51)
Other Hispanic	82.5	1.98 (0.63‐6.17)
Chinese	70.4	1.00 (0.68‐1.46)
Asian Indian	45.1	**0.34 (0.16‐0.76)**
Filipino	69.7	0.97 (0.33‐2.87)
Other Asian^a^	29.0	**0.17 (0.05‐0.55)**
Overall	69.3	–

OR, odds ratio; CI, confidence interval.

Bold values signify *P* < 0.05.

a Korean, Japanese, Vietnamese, and Other.

### 3.3 Sociodemographic factors

On univariable logistic regression, all variables except sex, birthplace, and education showed statistically significant associations with screening (Table 3). In the multivariable regression model—which excluded birthplace due to multicollinearity—screening was significantly more likely among individuals older than 65 years (OR 1.66, 95% CI 1.29‐2.14), primarily spoke Chinese at home (OR 2.52, 95% CI 1.13‐5.61), and were not part of the labor force (OR 1.60, 95% CI 1.06‐2.42). Screening was less likely among individuals who had lived in the US for less than 5 years (OR 0.44, 95% CI 0.22‐0.87), spoke an Asian Indian language at home (OR 0.19, 95% CI 0.05‐0.81), had a household income less than twice the federal poverty line (OR 0.64, 95% CI 0.49‐0.83), and resided in a borough other than Manhattan (OR 0.76, 95% CI 0.60‐0.95). Notably, when screening among Asian and Hispanic subgroups were considered in aggregate, neither group had a statistically significant difference compared to NHW.

**Table 3:** Association between sociodemographic factors and up‐to‐date colonoscopy

Variable	% with Up‐to‐dateColonoscopy	UnivariableOR (95% CI)	MultivariableOR (95% CI)
Age			
50‐64	64.0	Ref	Ref
65 and older	77.0	**1.89 (1.53‐2.33)**	**1.66 (1.29‐2.14)**
Sex			
Male	67.3	Ref	Ref
Female	70.4	1.16 (0.95‐1.41)	1.06 (0.86‐1.30)
Race/ethnicity			
White, non‐Hispanic	70.4	Ref	Ref
Asian/Pacific‐Islander	61.7	**0.68 (0.47‐0.96)**	0.54 (0.26‐1.09)
Black, non‐Hispanic	68.2	0.90 (0.69‐1.17)	1.06 (0.79‐1.44)
Hispanic	71.7	1.06 (0.83‐1.36)	1.00 (0.67‐1.50)
Other, non‐Hispanic	50.9	**0.44 (0.22‐0.85)**	**0.49 (0.25‐0.96)**
Birthplace^a^			
United States	70.1	Ref	–
Europe	69.0	0.95 (0.63‐1.42)	–
Asia	63.3	0.74 (0.53‐1.03)	–
Africa	59.2	0.62 (0.30‐1.28)	–
Caribbean	72.5	1.12 (0.86‐1.46)	–
Central/South America	66.1	0.83 (0.58‐1.18)	–
Other	51.4	0.45 (0.20‐1.02)	–
Home language			
English	69.3	Ref	Ref
Chinese	70.3	1.05 (0.72‐1.52)	**2.52 (1.13‐5.61)**
Indian	17.3	**0.10 (0.02‐0.35)**	**0.19 (0.05‐0.81)**
Russian	61.8	0.72 (0.43‐1.20)	0.67 (0.67‐1.23)
Spanish	73.2	1.21 (0.94‐1.56)	1.42 (0.90‐2.23)
Other	62.5	0.43 (0.45‐1.21)	0.94 (0.54‐1.61)
Time living in US			
Born in US	70.1	Ref	Ref
Lived in US <5 years	40.4	**0.29 (0.16‐0.53)**	**0.44 (0.22‐0.87)**
Lived in US 5‐9 years	58.5	0.60 (0.34‐1.06)	0.70 (0.67‐1.34)
Lived in US 10+ years	69.7	0.98 (0.80‐1.20)	1.15 (0.85‐1.56)
Education			
College graduate	71.1	Ref	Ref
≤High school graduate	68.8	0.90 (0.72‐1.12)	0.95 (0.71‐1.28)
Some college	66.0	0.79 (0.59‐1.05)	1.14 (0.85‐1.52)
Employment status			
Unemployed	55.1	Ref	Ref
Employed	65.9	**1.57 (1.08‐2.28)**	1.30 (0.87‐1.94)
Not in labor force	73.6	**2.26 (1.56‐3.28)**	**1.60 (1.06‐2.42)**
Income relative to poverty line			
≥200% of poverty line	72.1	Ref	Ref
<200% of poverty line	65.9	**0.75 (0.61‐0.91)**	**0.64 (0.49‐0.83)**
Borough of residence			
Manhattan	75.9	Ref	Ref
Other boroughs	67.4	**0.66 (0.53‐0.81)**	**0.75 (0.60‐0.95)**

OR, odds ratio; CI, confidence interval.

Bold values signify *P* < 0.05.

a Birthplace demonstrated multicollinearity with Time Living in US and was removed from the multivariable model.

## 4 DISCUSSION

In this analysis of the 2014 NYCCHS, we found substantial variation in CRC screening among Asian and Hispanic subgroups. In addition, age, language spoken at home, time living in the US, employment status, income, and borough of residence were shown to be independent predictors of screening.

The large and diverse population of NYC creates an ideal environment to study health inequities, and we were particularly interested in screening uptake among Asians and Hispanics. When survey respondents were broadly categorized as NHW, NHB, Asian, or Hispanic, screening uptake was 9% lower among Asians (unadjusted OR 0.68, 95% CI 0.47‐0.96) and 1% higher among Hispanics (unadjusted OR 1.06, 95% CI 0.83‐1.36) compared to NHW. Although the difference between Asians and NHW was not significant in the multivariable adjusted model, these results indicate screening uptake among Asians overall had again decreased after making gains from 2003 to 2013.

More importantly, when Asian and Hispanic subgroups were disaggregated, significant variation in screening uptake became apparent. Screening in Mexicans and Asian Indians were significantly lower than NHW on univariable regression, which indicate that these groups may benefit from targeted intervention to promote screening. In contrast, there was no statistical difference in screening between NHW and those of Puerto Rican, Cuban, Dominican, Central/South American, Chinese, or Filipino subgroups. Screening was also lower among Other Asians, but interpretation is limited by the fact that this was an aggregate group created to address small sample sizes in the individual subgroups. These findings provide further evidence of the value of collecting and analyzing disaggregated racial/ethnic data.

Our study adds to the limited available literature on CRC screening in different racial/ethnic subgroups. A recent study of the California Health Interview Survey, which collected biennial data on CRC screening from 2001 to 2009, also found that Chinese individuals had the highest screening among Asians in 2009.^17^ Filipinos and Asian Indians were found to have lower uptake, which corresponds to our findings. A separate analysis merging the 2001‐2005 California data found that Filipinos and Asian Indians were both less likely to undergo screening than NHW,^18^ whereas in our dataset only Indians had significantly lower screening than NHW. National data from the 2009‐2014 Medical Expenditure Panel Survey (MEPS) found that CRC screening prevalence was 62.3% for NHW, 55.0% for Filipinos, 50.9% for Chinese, and 48.6% for Asian Indians.^19^ Despite geographic and temporal differences between the 2014 NYCCHS and these other data sources, it is notable that Asian Indians consistently emerge as the Asian subgroup with one of the lowest CRC screening uptake in the US.

Screening data on Hispanic subgroups have previously been reported from the National Health Interview Survey (NHIS) and MEPS. The 2013 NHIS showed that Puerto Ricans had the highest uptake (56.8%), followed by Mexicans (44.6%), while Cubans and Dominicans (38.6%) had the lowest.^20^ An earlier analysis merging the 2000‐2006 NHIS and MEPS data also found Puerto Ricans had the highest uptake, but Mexicans had the lowest.^21^ Our data of Hispanic New Yorkers found the highest screening prevalence in Dominicans (75.3%) and Puerto Ricans (72.3%), while Mexicans (44.4%) had lowest prevalence. These results suggest Dominicans living in NYC may be distinct from those living in other parts of the US with respect to screening behavior. This could be related to differences within the communities, including contextual factors such as migration history, or the influence of external programs such as C5. On the other hand, our data confirms the national finding that Mexicans have one of the lowest CRC screening rates among Hispanics. To our knowledge, there was no systematic difference in outreach or implementation of interventions for Hispanic subgroups in NYC.

Older age and higher income are established predictors of CRC screening, but the associations with language spoken at home, time living in the US, employment status, and borough of residence are less well characterized. Home or preferred language and duration of US residence are commonly used as measures of acculturation. Most studies,^22^, ^23^, ^24^ but not all,^25^ have shown that a higher degree of acculturation correlates with CRC screening. With respect to duration of residence, our data found that recent immigrants who have lived in the US for less than 5 years were half as likely to have received colonoscopy compared to US‐born respondents. The likelihood of up‐to‐date colonoscopy increased with duration of residence, and immigrants who had lived in the US for more than 10 years had a non‐significantly higher uptake than those born in the US. Consistent with our unadjusted analysis showing Asian Indian ethnic subgroup was associated with being unscreened, those who spoke an Asian Indian language at home were one‐fifth as likely to be up‐to‐date with screening colonoscopy compared to English speakers. Although neither Chinese subgroup nor language was a predictor of screening on univariable analysis, individuals who reported speaking Chinese at home were 2.5‐fold more likely to have received colonoscopy than English speakers on multivariable analysis. Using backward stepwise regression, we determined that the addition of race/ethnicity to the model explained the large increase in the risk estimate for Chinese language. Being Asian was a predictor of being unscreened on univariable analysis but race/ethnicity was no longer significant in the multivariable model. A similar result was found in a racially/ethnically diverse screening trial in San Francisco, in which the strength of association between screening adherence and Chinese as the preferred language increased from unadjusted to adjusted analysis while the association with race/ethnicity diminished.^26^ This suggests that in areas with a large proportion of immigrants, such as NYC and San Francisco, language and limited English proficiency may serve as a better predictor of culturally mediated health behavior than aggregated race/ethnicity.

Compared to unemployed respondents, those who were not in the labor force had a 1.6‐fold higher likelihood of receiving colonoscopy. A 2014 survey of community health centers also found that individuals not in the labor force, who are primarily retired, were more likely to undergo screening even after adjusting for age and other factors.^27^ This likely indicates that retirees possess a combination of time, motivation, and financial stability that is especially conducive for completing CRC screening. In particular, access to Medicare has been identified as a consistent facilitator of CRC screening in older adults.^28^

NYC contributes more than 2% of all CRC deaths in the US,^29^, ^30^ and understanding which areas of the city are underscreened will allow for targeted intervention. Our analysis shows that even after adjusting for a number sociodemographic factors, residence in the outer boroughs (Brooklyn, Queens, The Bronx, Staten Island) was associated with a 25% lower odds of receiving colonoscopy compared to residence in Manhattan. The geographic disparity in screening may reflect both residual confounding by sociodemographics as well as differential access to screening services in various parts of the city.

Several limitations of our study should be noted. First, this was a single year cross‐sectional analysis, and consequently sample size was limited for certain analyses and important temporal trends could not be assessed. Pooling multiple years of data would also allow for greater power to analyze disaggregated race/ethnicity or individual boroughs in adjusted analyses, which may have greater policy impact. While our study used the available NYCCHS data, future prospective studies may consider oversampling Asian and Hispanic subgroups to gain additional insight into screening and other health behaviors. Second, because the NYCCHS only asked respondents about colonoscopy use, we do not have data on the usage of other screening tests such as fecal occult blood test or flexible sigmoidoscopy. Although the colonoscopy prevalence underestimates the true screening prevalence, the discrepancy in these two measures is attenuated by the fact that nationally 94% of individuals who are up‐to‐date on screening have received colonoscopy.^7^ Since C5 recommended colonoscopy as the preferred screening modality, the predominance of colonoscopy in NYC is likely similar to if not greater than the national rate. Third, Chinese was the only Asian language used to administer the 2014 NYCCHS, and the languages of other large subgroups such as Asian Indians and Filipinos were not represented. This may explain the finding that only 12 individuals in the sample reported an “Indian” language as their primary home language. If the survey used English rather than a preferred language for many Asian participants, then the accuracy of data collected on those individuals with limited English proficiency could have been impacted.^31^ Finally, a study by Cole and colleagues observed that traditional surveillance methods, such as random‐digit‐dial telephone surveys, may underestimate risk among more hard‐to‐reach minority and immigrant populations in NYC.^32^

## 5 CONCLUSION

In summary, while NYC is making significant strides in increasing CRC screening, our study found that significant gaps and disparities remain for certain Asian and Hispanic subgroups, as well as recent immigrants, low‐income individuals, and individuals who live in boroughs other than Manhattan. These findings can inform public health efforts to increase CRC screening through programs targeting these under‐reached and underserved New Yorkers.

## AUTHOR CONTRIBUTIONS

Neelesh Rastogi: study concept and design, data analysis and interpretation of data; drafting of manuscript. Yuhe Xia: data analysis, biostatistics. John Inadomi: critical revision of manuscript. Simona Kwon: critical revision of manuscript. Chau Trinh‐Shevrin: critical revision of manuscript. Peter Liang: study concept and design, data analysis and interpretation of data; critical revision of manuscript.

## CONFLICT OF INTEREST

None.
